# Cornmeal-based artificial diet improves development and reduces rearing costs of *Spodoptera litura*

**DOI:** 10.3389/finsc.2025.1732857

**Published:** 2025-12-19

**Authors:** Aning Fan, Yibo Huang, Nipapan Kanjana, Hanzhang Wang, Jianjun Mao, Yuyan Li, Junjie Zhang, Lisheng Zhang

**Affiliations:** 1 Jilin Province Technology Research Center of Biological Control Engineering, Institute of Biological Control, Jilin Agricultural University, Changchun, China; 2 State Key Laboratory for Biology of Plant Diseases and Insect Pests, Institute of Plant Protection, Chinese Academy of Agricultural Sciences, Beijing, China; 3 College of Plant Protection, Shanxi Agricultural University, Taigu, Shanxi, China; 4 Program of Agriculture, Faculty of Agricultural Production, Maejo University, Chiang Mai, Thailand; 5 The National Engineering Laboratory of Crop Stress Resistance, School of Life Sciences, Anhui Agricultural University, Hefei, China; 6 College of Horticulture and Landscape Architecture, Tianjin Agricultural University, Xiqing, China; 7 Control (North) of Ministry of Agriculture and Rural Affairs, Shanghai Veterinary Research Institute, Chinese Academy of Agricultural Sciences, Shanghai, China

**Keywords:** artificial diet, production costs, mechanized propagation, *Spodoptera litura*, *Telenomus remus*

## Abstract

The tobacco cutworm, *Spodoptera litura* (Fabricius) (Lepidoptera: Noctuidae), is a major defoliating pest in East and South Asia and serves as an alternative host for the mass propagation of the parasitoid *Telenomus remus*. Therefore, optimizing the rearing cost of *S. litura* is critical for large-scale production to enhance efficiency and reduce costs. We evaluated 18 artificial diet formulations derived from a standard diet by incorporating corn flour and adjusting the ingredient ratios. The diets were grouped into high-, medium-, and low-cost categories, and their biological performance was assessed under controlled laboratory conditions (26 ± 1 °C, 70 ± 5% RH, and a16:8 h L:D photoperiod). Among the tested diets, formulation 15 produced the best results, yielding shorter larval (19.52 d) and pupal durations (10.46 d), higher pupal mass (500 mg), greater fecundity (2,658 eggs/female), and improved egg hatch (73.77%) compared with the control. Population parameters, including the intrinsic (*r*) and finite (*λ*) rates of increase, were significantly improved, whereas the mean generation time (*T*) and net reproductive rate (*R_0_*) decreased. Importantly, formulation 15 lowered rearing costs by 5.9% relative to the original diet. These findings demonstrate that replacing soybean flour with corn flour as the primary protein source provides a cost-effective and biologically robust diet for *S. litura*. This optimized diet will facilitate large-scale rearing of *S. litura* and mechanized production of *T. remus*, providing a model for reducing artificial diet costs in other insect systems.

## 1 Introduction

The fall armyworm (*Spodoptera frugiperda* J.E. Smith) (Lepidoptera: Noctuidae) is a polyphagous pest native to the tropical and subtropical regions of the Americas. Owing to its voracious feeding capacity, rapid reproduction, and long-distance migratory ability, it has emerged as a global threat to food security (1, 2). For example, *S. frugiperda* infestations caused a 34% reduction in maize production and losses of more than US $400 million after they were first found in Brazil in 2006 (3). Between 2016 and 2018, the pest spread across Africa, invading 44 countries and leading to annual maize yield losses of 8.3–20.6 million tons, corresponding to economic damage of US $2.5–6.2 billion (4, 5). By late 2018, *S. frugiperda* reached Asia, with China reporting infestations that rapidly spread to 26 provinces within five months, damaging more than 1.1 million hectares of crops (6, 7). Chemical pesticides remain the dominant control strategy, owing to their immediate effectiveness and ease of application. However, excessive pesticide has accelerated pesticide resistance in *S. frugiperda*, disrupted natural enemy populations, and caused environmental concerns (8–10). To achieve sustainable management, biological control has become a cornerstone of integrated pest management. More than 290 natural enemies of *S. frugiperda* have been documented worldwide, including the egg parasitoid *Telenomus remus* (Hymenoptera: Platygastridae), which exhibits strong reproductive potential, efficient host utilization, and promise for large-scale application (11–13). *T. remus* females can penetrate the protective chorion layers of *S. frugiperda* egg masses and parasitize inner eggs, enabling high levels of field parasitism (14, 15). Field trials in Venezuela showed that releases of 4,500–9,000 individuals per hectare achieved parasitism rates approaching 90%, while in Guizhou, China, parasitism rates reached 84–100% (16–19). These results highlight the potential of *T. remus* as a highly effective biocontrol agent against *S. frugiperda* (20, 21).

Although *Telenomus remus* shows great potential for biological control, its mass rearing on *S. frugiperda* hosts remains limited by difficulties in maintaining stable host populations. Cannibalistic behavior among late-instar *S. frugiperda* larvae disrupts colony sustainability, posing a major obstacle to the development of reliable rearing systems (22). Alternative hosts have been explored, including *Corcyra cephalonica*, *Sitotroga cerealella*, *Mythimna separata*, and *Antheraea pernyi* (23, 24). Among these, *Spodoptera litura* (Fabricius) (Lepidoptera: Noctuidae) has emerged as a promising substitute. Long-term laboratory assays have shown that *T. remus* reared on *S. litura* eggs retain stable morphology, fecundity, and life table parameters across 30 generations (25). In addition, *S. litura* is amenable to mass rearing and exhibits strong biological compatibility with *T. remus*. A major limitation, however, is the high cost of *S. litura* artificial diets, which hampers affordable parasitoid production (26). Artificial diets have been employed in insect rearing for over a century, with formulations ranging from leaf-based diets to macronutrient-optimized blends of corn flour, soybean meal, yeast, and wheat germ (27–30). For example, diets with 35% mulberry leaf powder increased larval survival to 74% (31).

A recent study adjusted macronutrient ratios by using a blend of corn flour, soybean meal, and yeast (2:1:0.8 nitrogen ratio) with sucrose as a carbon source, which shortened development time, improved pupation rates and pupal quality, and optimized adult sex ratios (32). While these diets can improve larval development, pupal quality, and adult performance, their reliance on costly ingredients limits scalability. Corn flour, in contrast, is abundant, cost-effective, and nutritionally rich, providing nitrogen, proteins, and essential amino acids suitable for herbivorous insects.

By incorporating corn flour as a primary ingredient and adjusting the proportions of other components, we aimed to reduce rearing costs while maintaining or improving host quality. Thereby enabling reliable, cost-effective mass propagation of *T. remus* for biological control of *S. frugiperda.*

## 2 Materials and methods

### 2.1 Insect rearing and maintenance

A laboratory colony of *Spodoptera litura* was established from individuals originally provided by the experimental base of the Institute of Plant Protection, Chinese Academy of Agricultural Sciences (Langfang, Hebei, China 39°32′18″N 116°41′01″E). The colony was maintained under controlled environmental conditions (26 ± 1 °C, 70 ± 5% relative humidity, and a 16L:8D photoperiod). Larvae were reared individually in glass tubes (7.5 cm height × 2.5 cm diameter) to prevent cannibalism. First-instar larvae (<24 h post-hatching) were used in all experiments. For adult mating and oviposition, adults were transferred into plastic containers (6.5 cm diameter × 12 cm height) lined with wax paper to provide an oviposition substrate and covered with mesh lids for ventilation. A carbohydrate source was supplied daily in the form of absorbent cotton saturated with a 20% (v/v) honey solution. Egg masses were collected each morning for subsequent experimental use.

### 2.2 Artificial diet formulation and preparation

Eighteen experimental diets were developed by incorporating corn flour into the standard formulation and adjusting the proportions of major components. Diets were categorized according to relative cost into three groups: high-cost (CK, 3, 6, 9, 12, 15), medium-cost (1, 4, 7, 10, 13, 16), and low-cost (2, 5, 8, 11, 14, 17). The composition of each formulation is provided in **Table 1** (33).

**Table 1:** 18 artificial diet formulations.

Diet formula	Maize powder(g)	Soybean powder (g)	Yeast powder (g)	Wheat bran (g)	Cost	Diet formula	Maize powder (g)	Soybean powder (g)	Yeast powder (g)	Wheat bran (g)	Cost
CK	0	100	40	60	42.32	9	60	40	40	60	39.8
1	0	100	20	80	36.68	10	60	40	20	80	35.15
2	0	100	0	100	31.04	11	60	40	0	100	30.04
3	20	80	40	60	41.82	12	80	20	40	60	40.32
4	20	80	20	80	36.18	13	80	20	20	80	34.68
5	20	80	0	100	30.54	14	80	20	0	100	29.04
6	40	60	40	60	41.32	15	100	0	40	60	39.82
7	40	60	20	80	35.68	16	100	0	20	80	34.18
8	40	60	0	100	30.04	17	100	0	0	100	28.54

The composition of vitamins and preservatives was as follows: vitamin C (4g), niptibet (2g), sorbic acid (2g), cholesterol (0.8g).

Prior to use, corn flour, soybean flour, yeast powder, and wheat bran were dried in a hot-air oven at 120 °C for 2 h. The requisite amounts of each ingredient were weighed and thoroughly mixed. Separately, 800 mL of distilled water was brought to boiling, agar was added, and the mixture was stirred until complete dissolution. The agar solution was then combined with the dry mixture. A secondary solution containing vitamin C, niacin, sorbic acid, and cholesterol dissolved in 200 mL distilled water was incorporated into the diet once the temperature decreased to ~40 °C. The prepared diet was dispensed into sterile containers, cooled to room temperature, sealed, and stored at 4 °C until required.

### 2.3 Assessment of larval development and survival

To evaluate the biological performance of *S. litura* on the different diet formulations, freshly laid egg masses from the same cohort were incubated in Petri dishes (100 mm diameter) lined with moist filter paper under controlled conditions (26 ± 1 °C, 70 ± 5% RH, and a 16:8 h light: dark photoperiod). Hatching was monitored twice daily.

Newly emerged larvae were transferred individually into glass tubes (2.5 cm diameter × 7.5 cm height) containing an excess of diet and sealed with sponge plugs. Diets were inspected daily, with replacement as necessary. Developmental parameters—including molting times, survival rate, pupation date, pupation rate, and pupal duration—were recorded. Sex was determined at pupation, and pupae were weighed using an electronic balance. Pupal viability was maintained by placing pupae on moist filter paper in Petri dishes until adult emergence. Each dietary treatment comprised five groups, with 20 repetitions per group and one larva per replicate.

### 2.4 Assessment of adult reproductive performance and longevity

Adults emerging from each diet treatment were observed daily to record reproductive output and longevity. Egg masses deposited on wax paper or mesh covers were collected, transferred to Petri dishes, and sealed with Parafilm. Ventilation holes were made along the sealing margin using insect pins.

For each female, total egg production, oviposition period, pre-oviposition period, and hatch rate were recorded until death. Additional parameters measured included emergence rate, sex ratio, and adult lifespan.

### 2.5 Statistical analysis

Biological traits—including developmental duration, larval mortality, pupal weight, pupation and emergence rates, female proportion, adult longevity, fecundity, oviposition duration, and pre-oviposition period—were analyzed using one-way analysis of variance (ANOVA), followed by Tukey’s honest significant difference (HSD) test at *P* = 0.05. Analyses were conducted using DPS, and all figures were generated in GraphPad Prism v8.0 (GraphPad Software, San Diego, CA, USA).

Population-level parameters, including the intrinsic rate of increase (*r*), finite rate of increase (*λ*), net reproductive rate (*R_0_*), and mean generation time (*T*), were estimated according to the age-stage, two-sex life table method implemented in TWOSEX-MSChart (34–37). To obtain robust estimates of means and standard errors, a paired bootstrap test with 100,000 iterations was applied to demographic parameters across diet treatments.

The age-specific survival rate lx refers to the probability of a population surviving from egg development to age x, where β is the number of stages:


$$lx=\sum_{j=1}^{\beta }sxj$$

Age-specific fecundity (*m_x_*) is the average number of eggs laid per individual surviving to age *x*, where *f_xj_*is Age-stage-specific fecundity (the number of parasitoids that emerged from parasitized eggs):


$$mx=\frac{\sum_{j=1}^{\beta }sxjfxj}{\sum_{j=1}^{\beta }sxj}$$

The age-specific reproductive rate *m_x_*refers to the average egg production of a population at age *x*:

Age-stage survival rate (*s_xj_*) is the probability of a newly laid egg surviving to age *x* and developmental stage *j*, where *n_o1_* is the number of newborns used for the life table and *n_xj_* is the number of surviving individuals at age *x* and stage *j*:

sxj is the probability of an individual surviving from hatching to age x stage j:


$$sxj=\frac{nxj}{n01}$$

The intrinsic growth rate rm refers to the maximum growth capacity of a population under conditions of suitable environment, adequate food, and exclusion of adverse factors:


$$\sum_{x=0}^{\infty }e\text{-r(x+1)}lxmx=1$$

The net reproductive rate R0 is the total number of offspring produced by an individual over its lifetime:


$$R0=\sum_{X=0}^{\infty }lxmx$$

The average generation time *T* is the time required to increase *R_0_* when the population reaches a stable age-stage distribution and growth rate:


$$T=\frac{lnR0}{r}$$

Finite rate of increase (*λ*) is the population growth rate as time approaches infinity and population reaches a stable age-stage distribution:

The annual growth rate λ is the growth rate over a fixed time period:


$$\lambda =e^{r}$$

## 3 Results

### 3.1 Diet-dependent variation in larval development of *S. litura*

The developmental duration of *S. litura* varied markedly among instar stages when reared on different artificial diets (**Tables 2**, **3**, **4**). Under high-cost diets, larvae fed the No. 9 formulation showed significantly prolonged development at the fourth and fifth instars compared with other diets, whereas overall larval duration was significantly reduced under formulations 3, 6, 9, 12, and 15 relative to the control (CK). The most pronounced growth promotion occurred with the No. 6 formulation, which shortened the larval stage to 18.35 days. For medium-cost diets, developmental differences were less pronounced. Apart from the first instar, larvae fed the CK, No. 1, 4, 10,13 and 16 formulations showed no significant differences. However, larvae fed the No. 10 formulation had longer fifth- and sixth-instar periods compared with CK. The longest overall larval stage was recorded on the No. 7 formulation (23.75 days), in contrast to 20.14 days in the CK group, suggesting that some medium-cost diets may delay development.

**Table 2:** Developmental duration of *Spodoptera litura* after feeding on high-cost artificial diet.

Developmental stage	Artificial diet formula						*F*	*df*	*P*
	CK	3	6	9	12	15			
1st instar larva	4.98±0.02a	3.38±0.32b	2.54±0.10d	2.76±0.04cd	2.85±0.07cd	2.37±0.11bc	43.601	6	<0.0001
2st instar larva	3.59±0.12a	2.20±0.22c	2.55±0.08b	2.37±0.09bc	2.56±0.06b	4.01±0.16bc	17.463	6	<0.0001
3st instar larva	2.31±0.03b	3.59±0.20a	2.22±0.12b	2.66±0.20b	3.83±0.31a	3.83±0.11a	12.685	6	<0.0001
4st instar larva	3.18±0.104c	3.70±0.23b	4.46±0.18a	4.44±0.16a	3.66±0.18b	4.25±0.21b	10.501	6	<0.0001
5st instar larva	4.23±0.15bc	3.76±0.09c	4.41±0.06b	4.93±0.25a	4.35±0.22b	2.08±0.12bc	5.81	6	<0.0001
6st instar larva	3.88±0.19c	2.33±0.13a	4.08±0.04bc	3.96±0.06c	3.37±0.07a	4.08±0.12bc	4.34	6	0.006
Larva	20.14±0.24a	18.95±0.11b	18.35±0.21c	19.12±0.17b	19.53±0.25b	19.52±0.19b	9.249	6	<0.0001

Data expressed as mean ± SE, and those followed by different lowercase letters in the same row indicate significant difference among different artificial diet formulas (Tukey's test, *P*<0.05).

**Table 3:** Developmental duration of *Spodoptera litura* after feeding on medium-cost artificial diet.

Developmental stage	Artificial diet formula							*F*	*df*	*P*
	CK	1	4	7	10	13	16			
1st instar larva	4.98±0.02a	3.41±0.04bc	3.48±0.08cd	3.36±0.17d	3.35±0.10cd	4.06±0.25b	3.38±0.13cd	16.859	6	<0.0001
2st instar larva	3.59±0.12b	2.92±0.05a	2.68±0.06b	2.77±0.66b	2.70±0.15b	2.74±0.08b	3.02±0.11b	13.748	6	<0.0001
3st instar larva	2.31±0.03b	2.82±0.06a	3.16±0.13b	3.39±0.17b	3.11±0.11b	4.06±0.19b	3.26±0.17b	21.339	6	<0.0001
4st instar larva	3.18±0.104b	3.68±0.62a	3.07±0.10b	3.52±0.18b	2.99±0.10b	3.80±0.28b	3.10±0.18b	28.737	6	<0.0001
5st instar larva	4.23±0.15b	3.33±0.09a	3.65±0.21b	4.60±0.21b	4.69±0.29b	4.03±0.21b	4.34±0.35b	20.098	6	0.015
6st instar larva	3.88±0.19c	7.02±0.06d	6.85±0.09b	7.65±0.29a	7.74±0.09a	7.29±0.20ab	7.20±0.20ab	46.1	6	<0.0001
Larva	20.14±0.24a	21.4±0.80b	21.54±0.34a	23.75±0.40a	22.1±0.84a	22.74±0.76a	22.64±0.55a	40.051	6	<0.0001

Data expressed as mean ± SE, and those followed by different lowercase letters in the same row indicate significant difference among different artificial diet formulas (Tukey's test, *P*<0.05).

**Table 4:** Developmental duration of *Spodoptera litura* after feeding on low-cost artificial diet.

Developmental stage	Artificial diet formula							*F*	*df*	*P*
	CK	2	5	8	11	14	17			
1st instar larva	4.98±0.02a	3.84±0.14bc	4.17±0.07c	4.17±0.07b	3.83±0.48bc	3.54±0.17bc	2.63±0.09d	10.937	6	<0.0001
2st instar larva	3.59±0.12a	5.51±0.70a	5.42±0.30a	5.42±0.30a	6.47±2.78a	2.90±1.86a	7.81±2.25a	15.184	6	0.316
3st instar larva	2.31±0.03bc	12.38±1.98a	9.75±2.21ab	9.75±2.21ab	0	0	10.60±6.72ab	153.788	6	0.01
4st instar larva	3.18±0.104b	12.52±1.66a	9.00±2.81a	9.00±2.81a	0	0	0	144.788	6	<0.0001
5st instar larva	4.23±0.15ab	9.40±2.89a	6.00±3.30ab	6.00±3.30ab	0	0	0	65.348	6	0.004
6st instar larva	3.88±0.19a	0.50±0.50ab	1.00±1.00ab	1.00±1.00ab	0	0	0	2.462	6	0.089
Larva	20.14±0.24a	15.28±2.73b	8.72±0.63c	8.72±0.63c	2.16±0.13d	1.21±0.29d	3.79±0.61d	250.247	6	<0.0001

Data expressed as mean ± SE, and those followed by different lowercase letters in the same row indicate significant difference among different artificial diet formulas (Tukey's test, *P<0.05*).

By contrast, low-cost formulations produced variable and often adverse effects. Larvae fed formulations 2, 5, 8, 11, and 17 had significantly prolonged second- to fifth-instar periods relative to CK, indicating nutritional inadequacy. In contrast, the sixth-instar period was shortened under these diets. Notably, larvae fed the No. 11, 14, and 17 formulations failed to progress beyond the third to sixth instars, with developmental duration recorded as zero, demonstrating severe inhibition of larval growth.

### 3.2 Diet effects on survival and biological traits of *S. litura*

**Figure 1:**
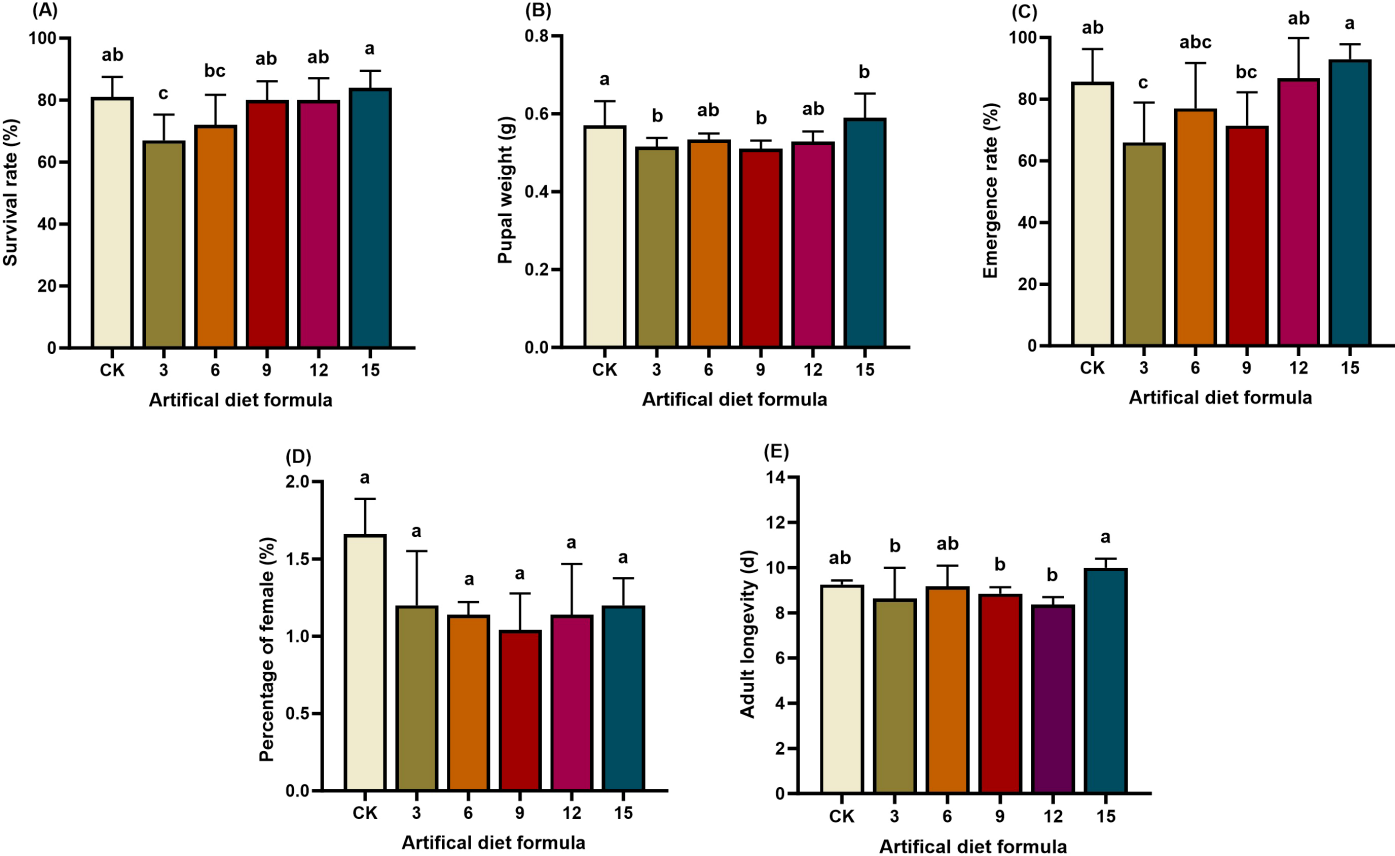
Biological characteristics of *Spodoptera litura* after feeding on high-cost artificial diet. **(A)** Survival rate; **(B)** Pupal weight; **(C)** Emergence rate; **(D)** Percentage of female and **(E)** Adult longevity. Pupal weight. Error bars represent standard deviation (*n* = 3). Different letters across the treatments indicate a statistically significant difference (*p<0.05*).

**Figure 2:**
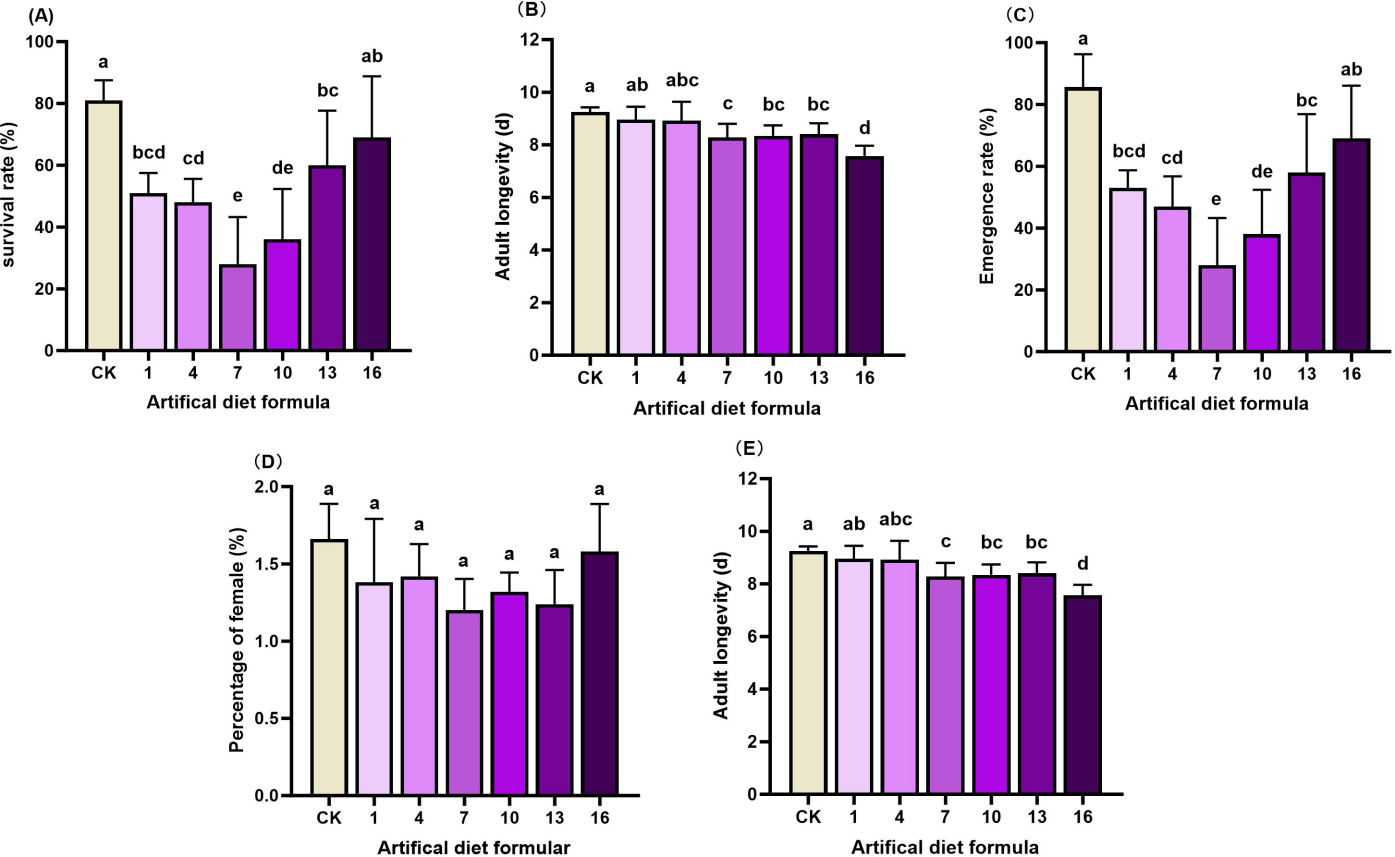
Biological characteristics of *Spodoptera litura* after feeding on medium-cost artificial diet. **(A)** Survival rate; **(B)** Pupal weight; **(C)** Emergence rate; **(D)** Percentage of female and **(E)** Adult longevity. Pupal weight. Error bars represent standard deviation (*n* = 3). Different letters across the treatments indicate a statistically significant difference (*p<0.05*).

**Figure 3:**
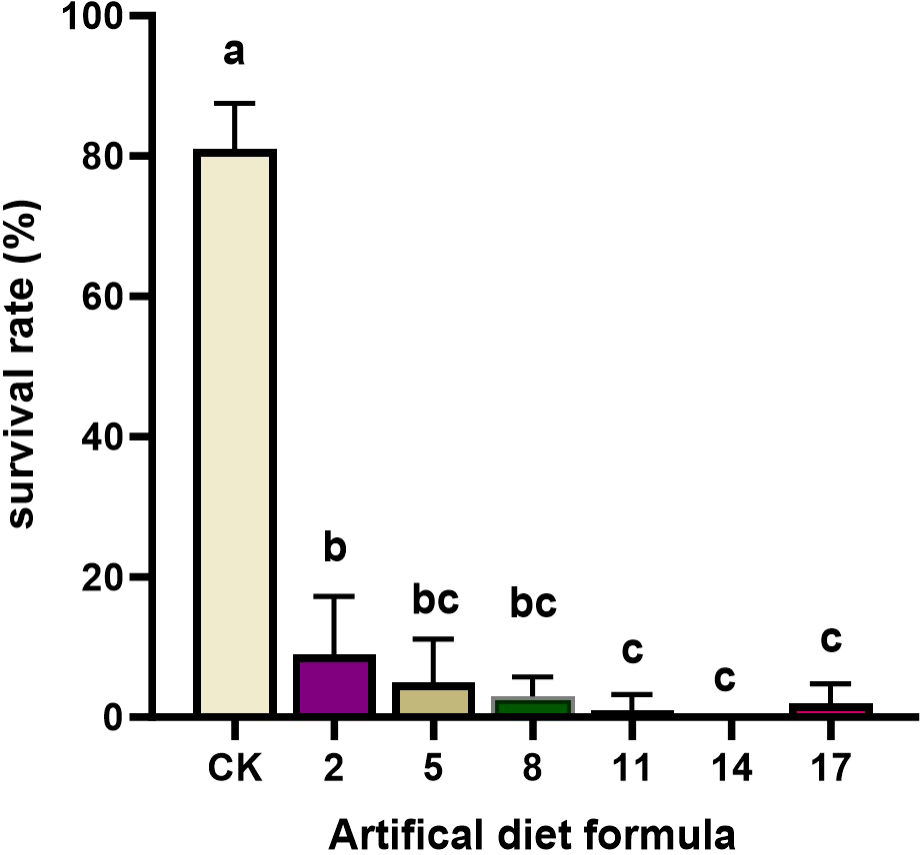
The survival rate and pupal weight of *Spodoptera litura* after feeding on low-cost artificial diet. Error bars represent standard deviation (*n* = 3). Different letters across the treatments indicate a statistically significant difference (*p<0.05*).

Survival and developmental success of *S. litura* were strongly influenced by diet composition (**Figures 1**, **2**, **3**). Under high-cost formulations, the No. 3 diet resulted in a reduced larval survival rate (67%), significantly lower than CK (81%) and formulations 9, 12, and 15 (80–84%). Correspondingly, pupation rate under the No. 3 diet was also depressed (66%), whereas pupal weight peaked under the No. 15 diet (0.59 g), significantly exceeding CK. In contrast, no significant differences were observed in adult lifespan or sex ratio across these diets.

Medium-cost formulations yielded more pronounced detrimental effects. Survival rates under diets 1, 4, 7, and 10 fell to 28–51%, markedly lower than CK (81%). Pupae from diets 10 and 13 were particularly small (0.426 g) compared with 0.44–0.57 g from other diets. Emergence rates under diets 1, 4, 7, 10, and 13 ranged from 28–58%, again significantly below CK (85.9%) and diet 16 (69%). Adult longevity was also compromised, with the CK group surviving longest (9.25d), compared with shorter lifespans under diets 7, 10, 13, and 16 (7.6–8.4d). As with high-cost diets, sex ratio remained unaffected.

Low-cost diets produced the most severe effects. Survival dropped drastically to 0–9% under diets 2, 5, 8, 11, 14, and 17, far below the CK survival rate of 81%. Larvae reared on these formulations failed to maintain normal growth and development, highlighting their unsuitability for laboratory rearing. Taken together, a comprehensive assessment of the developmental period, survival, pupation, and adult traits revealed that most high- and medium-cost formulations, as well as all low-cost formulations, failed to sustain healthy *S. litura* populations. Consequently, 16 formulations were eliminated. The No. 15 formulation emerged as the most promising candidate and was selected for further evaluation in subsequent rearing experiments.

### 3.3 Life table responses of *S. litura* to control and optimized diets

**Figure 4:**
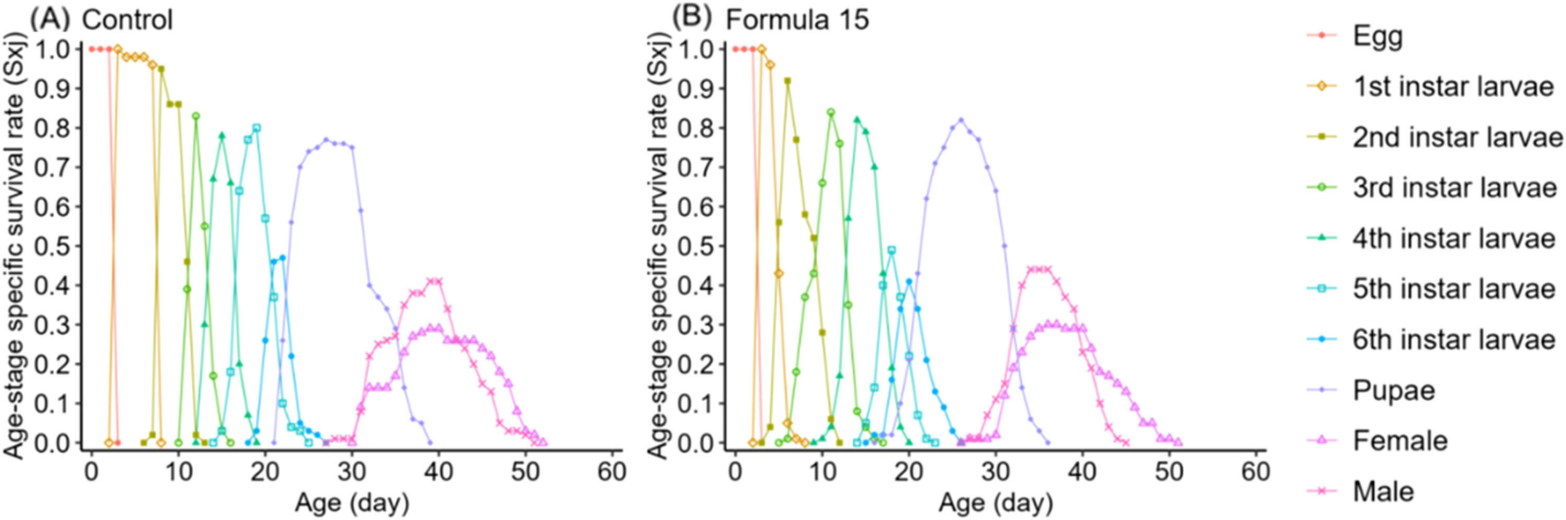
Age-stage specific survival rate (*sxj*) of *Spodoptera litura* feeding on the artificial diet of control **(A)** and formula 15 **(B)**.

**Figure 5:**
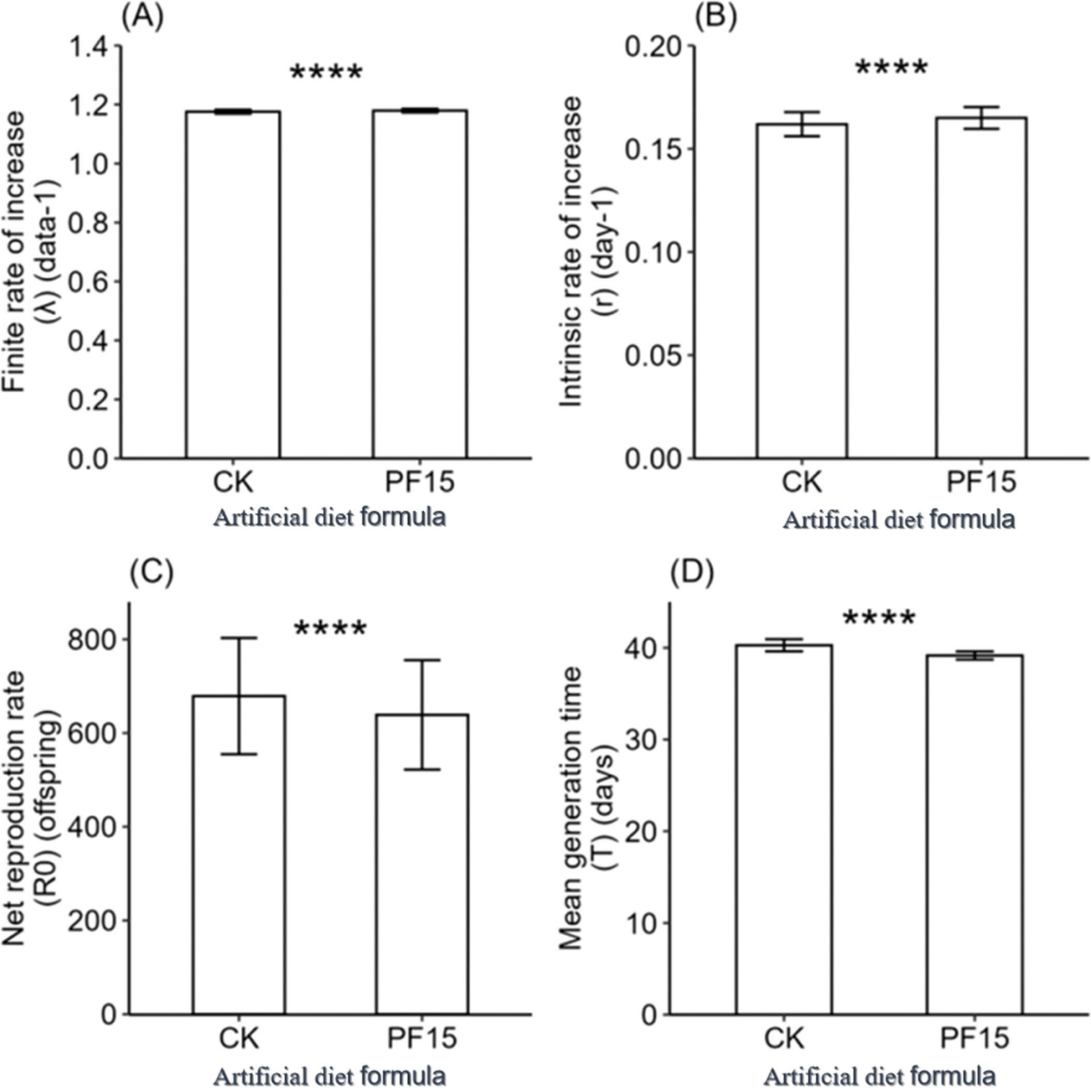
Population parameters of *Spodoptera litura* reared on control artificial diet and Formula 15diet. **(A–D)** represents finite rate of increase (*λ*), intrinsic rate of increase (*r*), net reproductive rate (*R_0_*), and mean generation time (*T*). Data are means ± SE. Asterisk in the figure indicate significant difference among different artificial diet formulas as determined by paired-bootstrap tests with 100,000 resampling (*P < 0.05, **P < 0.001, ***P < 0.001, ****P < 0.0001,**P < 0.01).

Life table analysis revealed marked differences in the developmental dynamics of *S. litura* when reared on the CK and No. 15 formulations (**Figures 4**, **5**). Overlapping developmental stages were evident under both diets, but the pupal stage varied slightly in duration (22–23d). Male adults consistently exhibited higher survival than females, with maximum survival rates of 41–44% compared with 29% in females.

**Figure 6:**
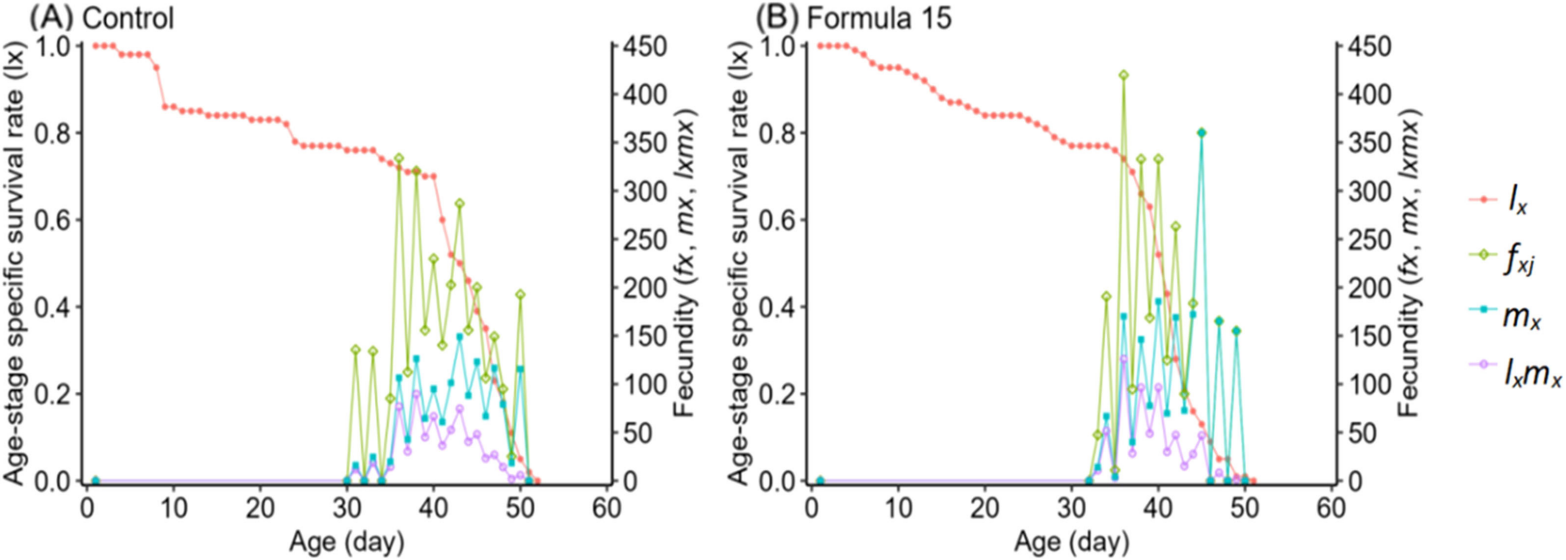
Female survival rate and fecundity of *Spodoptera litura* feeding on the artificial diet of control **(A)** and formula 15 **(B)**.

**Figure 7:**
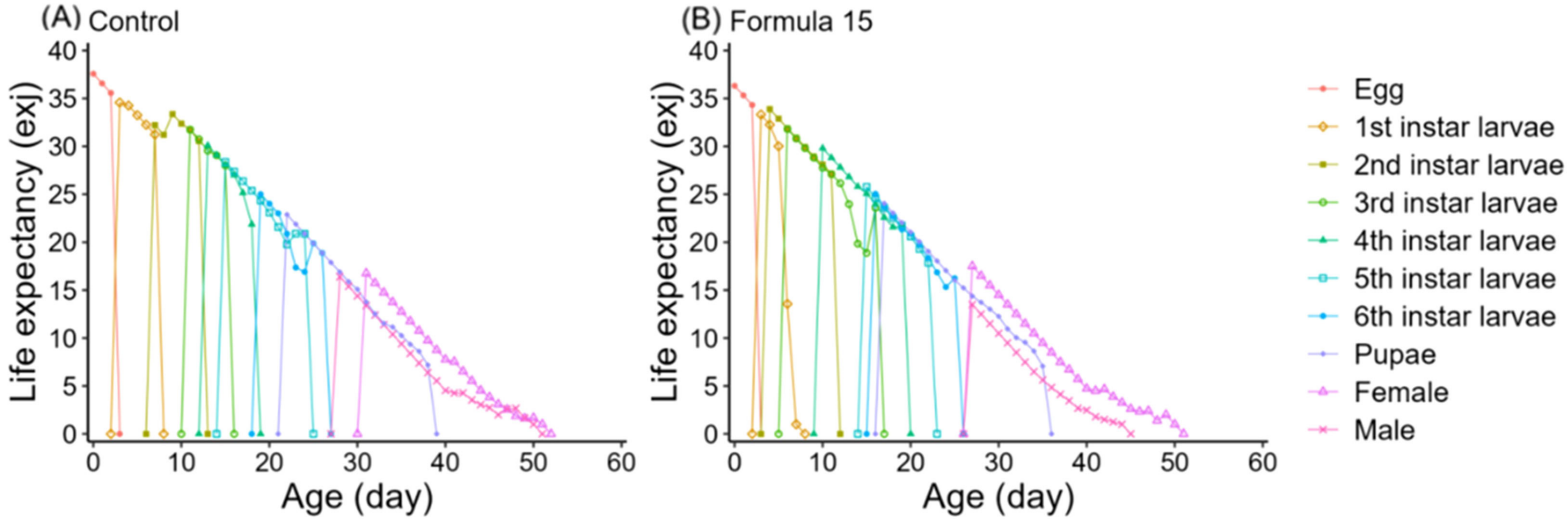
Age-stage specific expectancy (*exj*) of *Spodoptera litura* feeding on the artificial diet of control **(A)** and formula 15 **(B)**.

**Figure 8:**
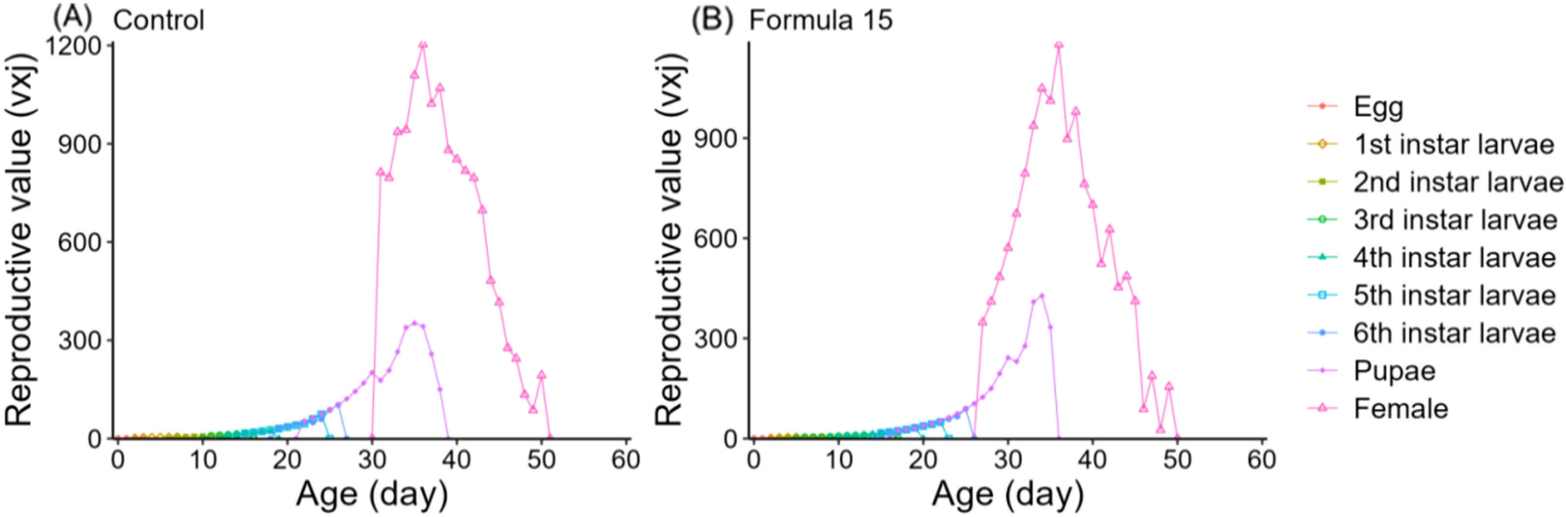
Age-stage specific reproductive value (*vxj*) of *Spodoptera litura* feeding on the artificial diet of control **(A)** and formula 15 **(B)**.

Age-specific survival (*l_x_*), fecundity (*f_xi_*, *m_x_*), and reproductive rate (*l_x_m_x_*) followed the expected trend of rising to a peak and then declining (**Figure 6**). However, peak fecundity and reproductive output were substantially higher in moths reared on the CK diet (149.16 eggs/female and 360.07 for *l_x_m_x_*) than on the No. 15 formulation (89.73 and 125.92, respectively). Female-specific fecundity also reached its maximum at day 36 under both diets, with higher values under the 15-formulation (419.73 vs. 333.69 eggs/female). Expected lifespan (*e_xj_*) gradually decreased over time (**Figure 7**). Initial values were comparable under CK (37.57d) and the No. 15 formulation (36.32d), though female longevity was slightly higher on the 15 diet (17.5 vs. 16.8d), while male longevity was reduced (13.5 vs. 16.4d). Reproductive value (*v_xj_*) followed the same rising–falling pattern, peaking at day 36 under both diets, with maximum values of 1203.36 d^-^¹ and 1180.70 d^-^¹ for CK and the 15 formulation, respectively (**Figure 8**).

Population growth parameters derived from TWOSEX-MSChart further underscored the superior performance of the No. 15 formulation (**Figure 5**). The intrinsic (*r*) and finite (*λ*) rates of increase were significantly higher than those of CK, while mean generation time (*T*) and net reproductive rate (*R_0_*) were reduced. These results indicate that, despite slightly lower fecundity values, the No. 15 formulation enhanced population growth potential and thus represents a nutritionally optimized artificial diet for *S. litura* rearing.

## 4 Discussion

Pests have developed resistance to a wide range of commonly used pesticides, particularly carbamates and pyrethroids, primarily due to chemical misuse. This resistance has significantly undermined the effectiveness of conventional control strategies (38). Consequently, employing natural enemies or biological control agents presents a more sustainable alternative, as it minimizes environmental harm and contributes to the maintenance of a healthier ecosystem (39).Artificial diets enable efficient insect mass rearing by reducing dependence on natural hosts and supporting controlled, sustainable rearing systems (40).

The present study demonstrates that optimizing artificial diet formulations can substantially enhance the growth, survival, and reproductive performance of *S. litura*, a preferred alternative host for the biological control agent *T. remus*, while simultaneously reducing rearing costs. Minimizing artificial diets costs is essential for large-scale industrial propagation, as diets must satisfy the nutritional requirements for normal insect development and maintain the appropriate balance of macronutrients for optimal metabolic function (41, 42).

The development of nutritionally balanced artificial diets has greatly facilitated the mass rearing of insects under controlled conditions, allowing detailed investigations into insect growth, behavior, and physiology (43). By incorporating corn flour as the sole plant-based component and adjusting the proportions of yeast powder and wheat bran, the high-cost 15-formulation diet achieved superior biological performance compared with the control, while reducing the diet cost by 5.9%. Specifically, larvae reared on this diet exhibited a larval period of 19.52 days, a survival rate of 84%, and a pupal weight of 0.59 g. The observed reproductive output (2,658.37 eggs per female) exceeded previously reported values for *S. litura* under standard diets (774 and 452 eggs per female), likely reflecting enhanced nutrient quality, controlled rearing conditions, and optimized experimental design (32, 44). These results underscore the feasibility of substituting soybean flour with cost-effective corn flour without compromising insect performance, consistent with earlier studies demonstrating the nutritional adequacy of corn-based diets for lepidopteran species (44).

Artificial diets are typically classified as pure, semi-pure, or practical feeds (45). While pure and practical diets are costly, labor-intensive, and may contain antinutritional compounds that hinder insect development, semi-pure diets offer a practical compromise, providing adequate nutrition for growth and reproduction while remaining suitable for large-scale propagation (46). Our findings indicate that semi-pure diets, when carefully balanced, can sustain high survival, rapid development, and robust reproductive performance in *S. litura*.

The present study also highlights the critical role of protein-to-carbohydrate ratios in larval development. Low-cost diets (100% wheat bran) resulted in larval mortality of 91–100%, whereas medium-cost diets (20% yeast powder + 80% wheat bran) produced extended larval periods, lower survival rates, reduced pupal weight, and shorter adult longevity. Conversely, the high-cost diet (40% yeast powder + 60% wheat bran) optimized these parameters, corroborating prior findings in cotton bollworm studies, where reduced yeast content delayed development and decreased survival (47). These results emphasize the importance of carefully calibrating nutrient ratios to support normal growth, development, and reproduction in *S. litura* (48, 49).

Corn flour, often underestimated in nutritional value, proved highly effective as the primary dietary component. Substituting soybean flour entirely with corn flour (100% ratio) significantly enhanced larval performance, confirming that corn-based formulations can satisfy the complete nutritional requirements of *S. litura* (41). In addition to ingredient optimization, reducing the proportion of costly components such as agar and vitamins or substituting cheaper alternatives could further decrease production costs without compromising performance (50, 51). From an applied perspective, the development of the 15-formulation diet offers a practical solution for cost-effective, large-scale rearing of *S. litura*, which in turn facilitates mass propagation of *T. remus* and advances biological control strategies. Nonetheless, caution is warranted to prevent inadvertent release of *S. litura* into agricultural fields, given its pest status (52). Ultimately, by examining developmental parameters, food utilization efficiency, and longevity across diets with differing protein levels, this study seeks to elucidate the phenotypic plasticity of *S. litura* (53).

Collectively, this study provides a framework for formulating low-cost, nutritionally optimized artificial diets for lepidopteran insects. The findings not only support basic research on insect physiology and population biology but also offer practical guidance for industrial-scale rearing and biological control programs, thereby contributing to more sustainable pest management strategies. Consequently, large-scale rearing of *S. litura* using this diet can effectively facilitate the propagation of the parasitoid wasp *T. remus*, supporting biological control programs and reducing reliance on chemical pesticides. This diet will further promote the successful, industrial-scale rearing of *T. remus* for use against *S. litura* in China, though more information is needed to optimize the mass-rearing system further.

## 5 Conclusion

*Spodoptera litura* (Fabricius) is a globally significant agricultural pest, and the development of cost-effective, nutritionally optimized mass-rearing systems is essential for sustainable pest management. In this study, we formulated an economically viable artificial diet for *S. litura* using corn flour as the sole plant-based component, combined with an optimized ratio of yeast powder and wheat bran. The high-cost 15-formulation diet not only supported robust larval growth, high survival rates, and enhanced reproductive performance but also reduced rearing costs by approximately 2.5 yuan per unit compared with traditional diets.

This optimized diet demonstrates that replacing soybean flour with corn flour is both feasible and advantageous, ensuring the nutritional requirements of *S. litura* are met while significantly lowering production costs. Consequently, large-scale rearing of *S. litura* using this diet can effectively facilitate the propagation of the parasitoid wasp *T. remus*, supporting biological control programs and reducing reliance on chemical pesticides. Furthermore, the findings provide a practical framework for developing low-cost, high-performance artificial diets for other lepidopteran species. Future research should evaluate the long-term effects of the 15-formulation diet over multiple generations and explore additional cost-reduction strategies, such as substituting or minimizing expensive components like agar and vitamins, to further enhance the sustainability of mass-rearing systems.

In summary, this study provides a robust and economically feasible solution for mass-rearing *S. litura*, with significant implications for integrated pest management and large-scale biological control applications.

## Data Availability

The original contributions presented in the study are included in the article/supplementary material. Further inquiries can be directed to the corresponding authors.
